# Improving primary palliative care – a Delphi consensus study on measures for general practice in Germany

**DOI:** 10.1186/s12875-021-01613-7

**Published:** 2022-01-17

**Authors:** Esma Sümeyya Bilgin, Rojda Ülgüt, Nils Schneider, Stephanie Stiel

**Affiliations:** Institute for General Practice, Hannover Medical School, Carl-Neuberg-Straße 1, 30625 Hannover, Germany

**Keywords:** Palliative care, Primary palliative care, Ambulatory care, Quality of care, Quality of health care, End-of-life care, General practitioner, General practice, Delphi study, Germany

## Abstract

**Background:**

The majority of severely ill and dying people in Germany can be administered primary palliative care (PPC) by general practitioners (GP). However, the current provision of PPC does not match the needs of the population. Although several public health strategies aim at strengthening the role of GPs in PPC provision, it remains challenging for GP teams to integrate PPC into their daily routines.

**Aim:**

A Delphi study with GPs was conducted to achieve consensus on specific measures for improving the integration of PPC into everyday GP practice.

**Methods:**

The study is part of the junior research project “Primary Palliative Care in General Practice” (ALLPRAX). After having developed, tested and evaluated 26 practical measures for GP practices to improve their PPC, a Delphi consensus study among GPs took place. In 2020, 569 GPs were asked to rate the relevance and feasibility of the measures on a 4-point Likert scale via an anonymous online questionnaire. Consensus was defined as a sum percentage of ‘strongly agree’ and ‘somewhat agree’ responses ≥75% after two rounds. Between these rounds, measures that were not consented in the first round were adapted in light of respondents’ free text comments and suggestions.

**Results:**

The response rate was 11.3% in round 1 (*n* = 64) and 53.1% in round 2 (*n* = 34). From the initial *n* = 26 measures, *n* = 20 measures achieved consensus and were included in the final intervention package. The consented measures pertained to four main topics: advance care planning with patients, consulting and informing patients and family caregivers, GP office organisation and continuing education. *N* = 6 measures did not achieve consensus, predominantly due to time and workload constraints.

**Conclusion:**

The consented measures provide valuable support to improve the provision of PPC by GPs. They can be used freely and flexibly, according to the needs of individual GP teams, and are thus suitable for implementation nationwide.

**Trial registration:**

The study was registered in the German Clinical Trials Register (Registration N° DRKS00011821; 4 December 2017; https://apps.who.int/trialsearch/) and the German Register of Health Care Research (Registration N° VfD_ALLPRAX_16_003817; 30 March 2017).

**Supplementary Information:**

The online version contains supplementary material available at 10.1186/s12875-021-01613-7.

## Background

In Germany in 2020, the total number of deaths was 985,620 [1]. Current international data show that “the proportion of individuals who died from diseases that indicate palliative care needs at the end of life ranges from 38 to 74% in European and non-European countries [2]. In consequence, the number of dying people potentially in need of palliative care in Germany might have ranged from 374,536 to 729,359 in 2020.

The organization of German palliative care involves a combined generalist-specialist model and offers inpatient and outpatient support. German estimates forecast that, at least 20% of people with cancer and 5% of people suffering from non-cancer diseases in their last phase of life require specialised palliative care by inpatient palliative care units or specialised palliative home care teams with 24/ call-on duty [3, 4]. The majority of all seriously ill and dying patients, and especially those suffering from non-cancer diseases, can be cared for through primary palliative care (PPC) in Germany. The main providers of PPC are primary care professionals, particularly general practitioners (GPs) and outpatient nursing services [5, 6]. Their main task is to recognize, prevent and reduce symptoms and problems of patients in need of palliative care. The aim is to provide PPC in a homely environment. Whenever patients’ symptoms and problems exceed the possibilities of PPC, specialised palliative care providers take over [7].

In Germany, the majority of GPs work full-time [8, 9] and GPs are mean 55.3 years of age [10]. Palliative medicine is a compulsory subject for all students in human medicine. The education guidelines for becoming a GP after studying medicine include basic information on palliative medicine and the treatment of patients with palliative care needs [11]. This allows GPs to offer generalist palliative care to their patients. GPs who want to extend their knowledge on palliative care and offer specialised palliative care can participate in a certified training including 40-h of theoretical courses and 120 h of supervised practical application [12].

Within the German federal state of Lower Saxony, a retrospective analysis of data from the statutory health insurance provider AOK on end-of-life care in the years 2016–2017 showed that only 28% of patients who died during this period received PPC prior to death [13]. This is far from the estimated need. At the same time, 9% of the seriously ill and dying patients received specialised palliative care –consistent with the estimated need [13]. In a retrospective cohort study by Ditscheid et al. (2020), use of different forms of palliative care (including PPC) in the 6 months prior to death were analysed, based on data from insured patients who died in 2016 [14]. The authors discovered that the number of patients receiving PPC was significantly lower than the reference figure from Radbruch et al., dating to 2014 [14, 15]. In contrast, the use of specialised PC covered the estimated need [14]. This emphasises the huge gap in provision between the different forms of palliative care, underlining the need for further insight into GPs’ barriers to administering PPC.

In focus group discussions within a German study by Krug et al. (2018), GPs identified communication with patients, family caregivers (FCs) and other service providers as a main barrier [16]. Specifically, the GPs cited difficulties understanding non-verbal messages from patients and FCs, as well as a lack of network support for adequate cooperation between GPs and other care providers [16]. Research by van Baal et al. (2020) highlighted further GP challenges, including time consuming bureaucratic procedures demanding intense and unrealistic personal commitments [13].

PPC is initiated during the very late stages of disease, shortly before death [13]. In particular, the early initiation of PPC for patients with (non-cancer) chronic diseases, either alongside or in lieu of curative therapy, is considered more challenging for GPs, relative to the delivery of PPC for cancer patients [17, 18]. Thus, delays in PPC provision are common, as a result of GPs’ prognostic uncertainties and difficulties predicting the disease progression [13].

Given this context, it is critical to determine what specific measures might help GPs deliver PPC (earlier) to seriously ill patients and integrate PPC more fully into their daily practice routines.

### Study aim

The present Delphi study aimed at achieving consensus within an expert group of GPs, with respect to the feasibility and relevance of *n* = 26 measures for PPC. The consented measures form a final evidenced-based intervention package for enhancing GPs’ PPC provision by overcoming common barriers [19].

### Previous work

Prior to the work presented here, three work packages of the umbrella project “Primary Palliative Care in General Practice” (ALLPRAX) were conducted:

I. In a first work package (November 2016–October 2017) four methods were used to explore barriers to and facilitators of GPs’ PPC provision [19–22].
II. In a second work package (November 2017–February 2019), a catalogue was developed based on *n* = 9 participatory action research group discussions with *n* = 28 participants in April 2019, including primary and specialised palliative and hospice care providers. *N* = 120 measures on structures and procedures how to improve GPs’ PPC provision were included [23, 24]. Some of these measures do not specifically address PPC patients but general routines in the GP practices. These measures aim at saving time and staff resources to be able to invest more capacities in palliative care delivery.
III. In the third work package (March 2019–October 2020), *n* = 8 participating GP teams were asked to select measures from the previously developed catalogue during two workshops. *N* = 35 measures were selected as demonstrating the greatest potential for improving PPC provision [24]. Material such as assessment sheets to implement these measures was developed and provided by the research team. Over a period of 4 months, the GPs and their teams implemented and assessed the selected measures. *N* = 26 measures were positively evaluated and included in the Delphi study at hand.

## Methods

All methods were carried out in accordance with relevant guidelines and regulations.

### Study application

**Fig. 1:**
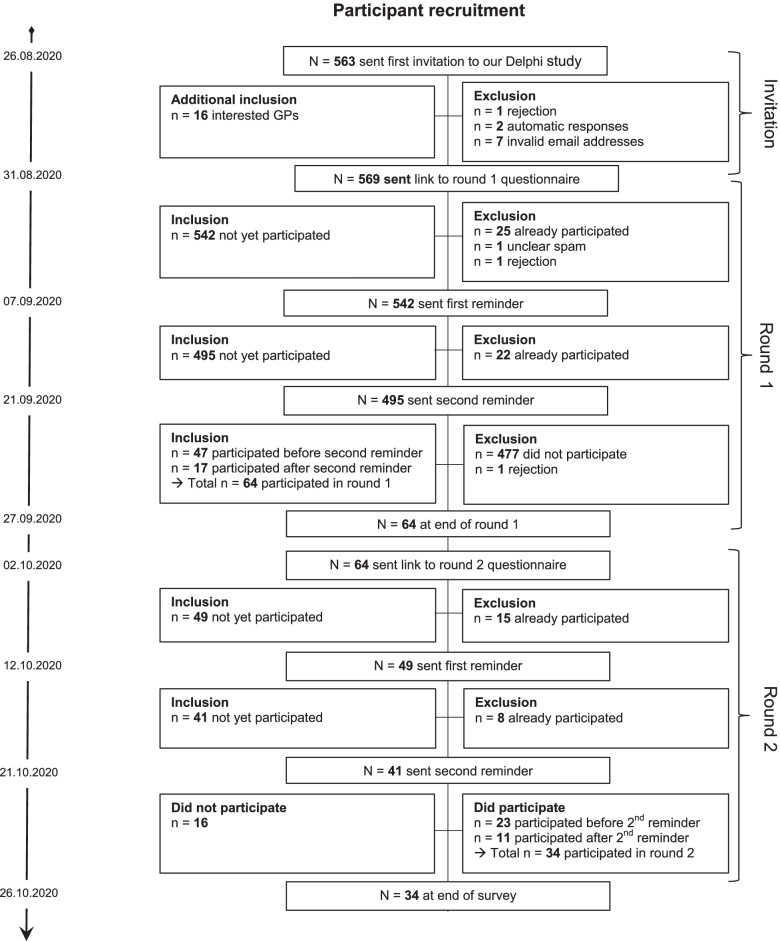
Participant recruitment: Preparation, round 1 and round 2

At the end of August 2020, the study team contacted *n* = 563 GPs working in Lower Saxony/Germany via email, to announce and provide information on a forthcoming Delphi study within the ALLPRAX project and invite them to take part. This number covers 10.86% of the total number of GPs working in Lower Saxony (*n* = 5185 in 2018) [10]. All of the recipient GPs had previously collaborated with our institute, with respect to research and/or education. After taking into account automated and individual responses, the email distribution list was modified and the expert group was formed (see Fig. 1).

Pre-test: The online questionnaire was designed using the EvaSys software. It included *n* = 26 measures pertaining to: (I) advance care planning with patients, (II) consulting and informing patients and FCs, (III) GP office organisation, (IV) continuing education and (V) cooperation with other service providers (see Table 1). Before sending the online questionnaire to the experts, we conducted a trial run with six participants, including members of the research team and other researchers from our institute. We then adjusted the questionnaire on the basis of their suggested changes, regarding technical problems and the clarity of terms.

**Table 1:** Main content domains of the specific Delphi study measures

	Domain	Items
I	Advance care planning with patients	5
II	Consulting and informing patients and FCs	5
III	GP office organisation	9
IV	Continuing education	5
v	Cooperation with other service providers	2
Overall		**26**

Delphi round 1: In September 2020, an email with detailed information about the survey and an individual link to participate in the first Delphi round was sent to the final study sample of *n* = 569 GPs. The round remained open for 4 weeks. Two reminder emails were sent to non-respondents in weeks 2 and 4.

Each expert was asked to anonymously rate each measure on a 4-point Likert scale ranging from 1 (fully agree) to 4 (fully disagree) [25, 26], with respect to two criteria:

1. relevance in the context of PPC at GP offices (“I (fully) (dis) agree to the relevance of …” ); and
2. feasibility in the context of PPC at GP offices (“I (fully) (dis) agree to the feasibility of …” ).

A 4-point Likert scale was chosen following the test construction criteria to avoid participants’ tendencies towards the middle of a scale in their response behaviour. Because of that, a tendency in terms of pro or contra to each measure was gained from each participant. Whenever participants responded ‘rather disagree’ (3) or ‘fully disagree’ (4), with respect to either relevance or feasibility, they were offered the opportunity to elaborate on their rating and provide suggestions for improving the measure in a free text field.

Individual measures were considered consented when the summarised percentages of ‘rather agree’ and ‘fully agree’ were > 75% for both relevance and feasibility. A 75% cut-off was defined following current Delphi study examples from the literature that state that this is the most common level for a definition of consensus [27]. Once this criterion was met, the measures were included in the final intervention package. Any measure that did not achieve consensus was adapted in line with the experts’ comments and included in the second Delphi round.

Delphi round 2: GPs who had responded to the first Delphi round were invited to participate in the second Delphi round in October 2020. This round remained open for 3 weeks, with reminder emails sent in weeks 2 and 3. The Delphi study closed at the end of October 2020 (see Fig. 1). GPs who participated in both rounds were offered the opportunity to voluntarily take part in a raffle at the end of the study, through which three GPs had the chance to win a 50€ voucher for the online shop “amazon”.

Informed consent was obtained from the participants prior to study participation in the online questionnaire.

### Socioeconomic questionnaire

At the end of each Delphi round, participants were asked to provide data on their age, gender, years of professional experience, completion (or not) of certified training in palliative care, office location (i.e. urban vs. rural), number of doctors in the practice and number of physician assistants in the practice. Only the sociodemographic data of participants in the second round were evaluated.

### Statistical evaluation

For the calculation of the descriptive statistics (i.e. means, standard deviations, medians) and absolute numbers and percentages, SPSS software version 25 [28] was used.

## Results

### Response rate

We sent out an initial information to *n* = 563 GPs and introduced the concept and aim of our project to announce that an invitation to participate in a study will follow shortly. In response to this information, we received *n* = 1 wish not to participate, *n* = 7 automatic replies indicating an invalid email address and *n* = 2 automatic replies indicating that emails were not being read. Apart from these 10 responses, we received *n* = 16 additional emails from interested GPs who heard about our upcoming survey without receiving our first email invitation. After incorporating all replies, we sent out the email invitation including a link to the online questionnaire to *n* = 569 GPs. (see Fig. 1).

The participation rate in the first round was 11.3% (*n* = 64). Of these 64 GPs from the first round, 53.1% (*n* = 34) participated in the second round. The participation rate of both rounds was 5.6% (*n* = 34 out of *n* = 569).

### Participants’ socioeconomic background (see Table 2)

The GPs who participated in Delphi round 2 were mean 55.5 years old (range: 36–71 years) with an average of 25.8 years of work experience (range: 3–43 years). With respect to gender, 71% of the participants were male. A small majority (51%) had received certified training in palliative care. The median number of working GPs in their practice was 2, and the mean number of physician assistants in their practice was 4. Regarding office location, 56% were located in urban areas whereas 44% were located in a rural area.

**Table 2:** Demographic characteristics of participants in round 1 and round 2

Socioeconomic characteristics		Round 1	Round 2
Gender distribution [%]	Female	39	29
	Male	61	71
Certified training in palliative care [%]	Yes	47	51
	No	53	49
Location of the office [%]	Rural	39	44
	Urban	61	56
Age [years]	Median	56	58
	Mean	54.69	55.52
	Range	34–71	36–71
Years of experience [years]	Median	25	26
	Mean	25.97	25.76
	Range	8–47	3–43
Number of doctors in the office [number]	Median	2	2
	Mean	2.97	3
	Range	1–15	1–14
Number of medical assistants in the office [number]	Median	5	4.5
	Mean	6.41	8.7
	Range	1–70	2–75

### Measure overview

In the first round, 11 out of the 26 measures achieved consensus. In the second round, 9 of the remaining 15 measures achieved consensus. 6 of the initial 26 measures did not achieve consensus in either round (see Table 3). Please also see the online Additional file 1 for a detailed illustration of the exact percentages for relevance and feasibility, comments from the participants and changes in wording each round for each measure.

(I) Advance care planning with patients

**Table 3:** Overview: Wording of measures in both rounds, round of consensus and percentages for relevance and feasibility (FCs = family caregivers, n.c. = no consensus reached, PPC = primary palliative care, GP = general practitioner, ECG = electrocardiography)

	Final version in round	Round 1 wording	Round 1 relevance [%]	Round 1 feasibility [%]	Round 2 wording	Round 2 relevance [%]	Round 2 feasibility [%]
I. Advance care planning with patients							
M1	2	One selected member from the general practice team conducts a structured first assessment with patients receiving PPC and their FCs.	64.1	57.4	The general practice team conducts a structured first assessment with patients receiving PPC and their FCs.	85.3	76.4
M2	2	The general practice team develops, in cooperation with patients receiving PPC, an emergency dataset. The dataset is saved in the general practice and/or perspectively on the electronic health card.	78.2	55.6	The general practice team develops, in cooperation with patients receiving PPC, an emergency dataset. The dataset is saved in the general practice and distributed to the patient.	97.0	84.9
M3	2	GPs identify patients who might benefit from PPC using structured tools (e.g. SPICT).	68.8	75.8	Drawing on their personal experience with the support of structured tools (e.g. the Supportive and Palliative Care Indicators Tool), general practitioners identify patients who might benefit from PPC.	81.8	78.8
M4	1	A filled in and up-to-date crisis paper sheet is kept clearly visible at the palliative patient’s home (e.g. next to his or her bed).	95.4	91.8			
M5	1	The general practice team maintains a copy of each palliative patient’s crisis sheet in the office.	84.1	85.7			
II. Consulting and informing patients and FCs							
M6	1	Telephone numbers of emergency contacts and proxies, as selected by the patient, are saved in the practice computer system.	95.3	95.2			
M7	2	GPs distribute leaflets with information about palliative care to patients, who could benefit from PPC as well as their FCs.	79.3	83.9	In addition to making personal consultations, GPs distribute leaflets with information about palliative care to patients who could benefit from PPC, as well as their FCs.	85.3	85.3
M8	2	GPs distribute leaflets with internet resources or information on advance directives and/or health care proxies to patients with a potential palliative disease progression.	71	79	In addition to making personal consultations, GPs distribute leaflets with internet resources or information on advance directives and/or health care proxies to patients with a potential palliative disease progression.	100	97.1
M9	1	One selected member of the general practice team informs palliative patients about the possibility of a release of confidentiality restraints between involved service providers, to facilitate cooperation and communication between the practice team and other service providers.	76.2	75.4			
M10	1	GPs inform all patients and their FCs about the standby service outside of regular practice hours.	76.6	87.1			
III. GP office organisation							
M11	1	Physician assistants refill the home visit bag directly after a completed home visit.	80.9	76.6			
M12	2	One selected member from the general practice team coordinates the care of patients receiving PPC	68.8	68.8	The general practice team coordinates in joint consultations around the care of patients receiving PPC. If possible/necessary, cooperation with palliative support centres or palliative care teams is initiated.	88.2	85.3
M13	2	Medical assistants are responsible for updating the medication plans of all patients.	52.3	47.4	GPs are responsible for updating the medication plans of patients receiving PPC and reviewing indications on a regular basis.	97.1	97.0
M14	n.c.	Continuing education courses offered internally in general practices are made accessible to further cooperating care providers and/or interested parties, such as home health care services and will be actively advertised.	63.5	50.8	Continuing education courses offered internally in general practices are made accessible to further cooperating care providers and/or interested parties, such as home health care services.	64.7	51.5
M15	1	General practices provide written instructions and options for looking up instructions for rare tasks (e.g. professional association case procedures, rehabilitation requests).	77.8	75.4			
M16	1	When registering patients in the general practice, physician assistants ask patients about their preferences, document their complaints and, if necessary, initiate the first treatment steps (e.g. direct the patient to a specific room for ECG).	92.2	91.9			
M17	2	Provide physician assistants with an undisturbed environment for documentation during working hours.	90.6	59.7	If the practice rooms allow it, provide physician assistants with an undisturbed environment for documentation during working hours.	97.1	82.4
M18	1	The practice team holds regular team meetings.	96.9	92.1			
M19	2	For admission requests to hospices or palliative care units, general practice teams use a standardised fax template.	67.2	80.4	For admission requests to hospices or palliative care units, general practice teams use a standardised fax template, which can be supplemented with individual annotations. Where necessary, personal contact is initiated to discuss more complex patients.	91.2	85.3
IV. Continuing education							
M20	1	GPs offer internal courses for their physician assistants on e.g. hygiene standards.	89.0	81.9			
M21	1	GPs realize physician assistants’ continuing education requests in a timely manner.	93.7	81.0			
M22	n.c.	Members of the practice team participate in internal courses on palliative care and end-of-life communication.	82.5	72.1	Members of the practice team participate in internal or external courses on palliative care and end-of-life communication.	87.9	69.7
M23	n.c.	Members of the practice team participate in internal courses on advance directives and advance care planning.	59.3	60.6	Members of the practice team participate in internal or external courses on advance directives and advance care planning.	76.4	67.6
M24	n.c.	Physician assistants accompany GPs on home visits to patients receiving PPC, to gain hands-on practical experience in palliative care.	78.1	61.1	If staffing capacity allows, physician assistants accompany GPs on home visits to patients receiving PPC, to gain hands-on practical experience in palliative care.	91.2	70.6
V. Cooperation with other service providers							
M25	n.c.	Physician assistants complete an internship at a palliative and/or hospice care provider for one month.	57.2	31.2	If staffing capacity allows, physician assistants complete an internship at a palliative and/or hospice care provider (e.g. for one week).	85.3	46.9
M26	n.c.	Before a GP is temporarily substituted with another, patients with high demand for care are discussed via a medical information handover during joint home-visits, with a corresponding exchange of documents.	90.6	64.5	Before a GP is temporarily substituted with another, patients with high demand for care are discussed via a medical information handover over the telephone, with a corresponding exchange of documents.	97.0	73.5

All five measures on (I) advance care planning with patients were consented in round 1 (M4, M5) or in round 2 (M1, M2, M3). These measures include a structured first assessment on e.g. symptoms and problems, medication of patients receiving PPC (M1) as well as the implementation of an emergency dataset (M2) containing e.g. family caregiver contact data. Additionally, use of a structured tool (e.g. the Supportive and Palliative Care Indicators Tool [29] to identify patients who might benefit from PPC (M3) were consented as a helpful addition to personal experience. Furthermore, our participants agreed on conducting a palliative ‘crisis sheet’ in the office and at the patient’s home (M4, M5), which record PPC patients’ preferences for emergency situations. For this, a common language should be used so they can be easily understood by all care providers, as expressed by our participants.

(II) Consulting and informing patients and FCs

Measures on (II) consulting and informing patients and FCs were all consented either in round 1 (M6, M9, M10) or in round 2 (M7, M8). They include the placing of telephone numbers for emergency contacts and proxies (as selected by the patient) close to the patient’s bedside, as well as saving this information in the practice’s computer system (M6). Furthermore, leaflets about palliative care (M7) and about advance directives and/or health care proxies (M8) as a complementary resource to personal consultations, were consented by our participants. In addition, our GPs agreed on informing patients about the medical standby service outside of regular practice hours (M10), but suggested an access to a more specialised emergency network for patients receiving palliative care.

(III) GP office organization

Regarding the measures on (III) GP office organization, four out of nine measures were consented in round one (M11, M15, M16, M18). They include the refill of home visit bags (equipped with medical devices and medicine) by physician assistants (M11) immediately after home visits to prepare for upcoming palliative care home visits, even though *n* = 4 GPs preferred to refill these bags themselves. Furthermore, GP offices should provide written instructions and options for looking up rare tasks (e.g. professional association case procedures, rehabilitation requests) (M15). In addition, physician assistants should document preferences (e.g. which doctor has to be consulted in the practice) and current subjective complaints (e.g. having cough) of all patients entering the practice and initiate first treatment steps if needed at each of their practice visits (M16), having difficulties referring to e.g., patient concerns and data privacy in mind, as underlined by our GPs.

Four out of the remaining measures were further consented in round two. They include joint consultations of the general practice team for coordinating around the care of patients receiving PPC (M12). *N* = 3 out of 64 experts suggested further cooperation with palliative care support centres or palliative care teams for more complex patients, as these institutes deliver specialised palliative care and can support GPs with their expertise [30]. Another measure, our GPs agreed on, is the updating of medication plans of patients receiving PPC and reviewing indications on a regular basis (M13). This measure made medical assistant responsible for this task initially, but was heavily criticised by the participants, who argued that the responsibility for changes to the medication plan should lay with doctors.

Furthermore, an undisturbed environment (e.g. a back office) for documentation during working hours should be provided for physician assistants (M17), even though our GPs expressed doubts with respect to space and time constraints. The measure on using a standardised fax template for admission requests to inpatient hospices or palliative care units, which should include additional space to record individual information, was consented by our GPs (M19). Where necessary, follow-up contact via telephone should be initiated to discuss the takeover of a patient.

The only measure our participants did not agree on in this category is to make internal education courses accessible to cooperating care providers and/or interested parties, such as home health care services (M14). A lack of time and space in GP offices was raised as a limiting factor.

(IV) Continuing education

Regarding the measures on (IV) continuing education, two measures were consented in round one (M20, M21). They include internal courses for physical assistants (M20) and realizing physician assistants’ continuing education requests in a timely manner (M21), both realized by the GPs. The two measures pertaining to continuing education courses for the practice team on important PPC topics (M22: palliative care and end-of-life communication; M23: guidance on advance directives and advance care planning) were not consented. Time and staff constraints due to high workloads and a lack of offers for external courses on palliative care topics were the GPs’ main contra arguments, generating low feasibility ratings. GPs assessed the measure of being accompanied by physician assistants on home visits to patients receiving PPC (M24) as relevant for improving their team’s hands-on practical experience in palliative care. However, staff and time constraints resulted in low feasibility. Furthermore, one expert raised that hands-on palliative care experience is already incorporated in the training for specialised medical care assistants.

(V) Cooperation with other service providers

Neither in round one, nor in round two were the two measures on (V) cooperation with other service providers consented (M25, M26). Participants’ main reasons for a low feasibility were a lack of time to invest into multidisciplinary communication and a lack of missing cooperating partners. They include an internship for physician assistants with a specialised palliative care provider (e.g. palliative care unit or inpatient hospice) for 1 week (M25). Our GPs argued, that they could not do without a physician assistant for an extended period of time. In addition, they pointed out that financing this time off would be challenging. They further referred to the imbalance between the number of patients receiving PPC and the number of patients without palliative care needs in the GP office. The other measure states that patients with high demand for care should be discussed in a medical information handover over the telephone with a corresponding exchange of documents before a temporarily substitution of a GP with another (M26). Although participants agreed on the relevance of a medical information handover between two GPs prior to a temporary substitution (M26), this measure as evaluated as too time-consuming and not feasible in practice. One GP advocated for the development of a specific interface to simplify communication about palliative care patients. This interface could be a digital network between GP practices to hand over patient data and to connect external partners in palliative care into one documentation system shared by all involved caregivers, we assume.

## Discussion

After having developed, tested and evaluated 26 practical measures for GP practices to improve their PPC, a Delphi consensus study among GPs took place. At least 20 of these measures achieved consensus on relevance and feasibility and were included in a final intervention package.

The main results showed an acceptance of measures focussing on strategies to prevent and prepare for emergency situations in PPC patients. In a study by Wiese et al. (2008), specific preparation for emergency situations contributed to reducing the hospitalisation rate amongst patients receiving palliative care [31]. Thus, a better and more frequent implementation of this measure in GP offices may improve the provision of PPC.

The expert group emphasised the importance of personal experience in identifying patients with palliative care needs. However, they also confirmed the value of using systematic tools such as the Supportive and Palliative Care Indicators Tool to support their professional assessment. Differences among the participants in terms of experience in palliative care are likely to have influenced this result. The more experience a GP has in palliative care, e.g. through a certified training, the more this can support their ability in identifying palliative needs. A previous study (2019) by our research team found that uncertainties regarding the identification of patients and the timing and circumstances under which PPC should be implemented were the main factors contributing to delays in the provision of PPC [21]. The expert-consented measure in the present study combines GPs’ preference for flexibility and acknowledgement of their personal experience with a guide to support their decisions. Thus, this measure has the potential to increase GPs’ early integration of PPC.

The participating GPs agreed upon measures involving FCs into the treatment programme. Due to the overwhelming situation of palliative care patients, their FCs often suffer from significant distress (including sadness and exhaustion), as emphasised by Ullrich et al. (2017) [32]. This may lead to a severe reduction of their wellbeing. The early integration of FCs in consultations and an additional focus on their wellbeing would play a key role in maintaining FCs’ physical and psychological health. This is essential for maintaining their quality of life and allowing them to remain to offer informal care to patients with palliative needs [32]. Accordingly, the consented measure has the potential to highlight the importance of FCs as a patient themselves and as an integral part of PPC and thus ease FCs’ physical and psychological distress.

Against expectation, measures involving cooperation with other care providers were not included in the final intervention package, even though cooperation with colleagues was assessed as relevant for the improved provision of PPC, but not feasible due to lacking time and staff resources. These arguments are sometimes experienced as “easy excuses” with short term view, but do not shed a light on the necessity to solve a long-term problem with a rather small investment that can save a lot of time later. A German study by Peter et al. (2020) shows that a lack of multidisciplinary cooperation between GPs and other palliative care providers is a main barrier for GPs’ provision of high-quality PPC [33]. In general, not only the willingness of GPs to contribute to intervention studies is rated low [34], but also the feasibility of intervention measures in many fields of GP work and in GP offices [35].

Same criticism is true for the non-consensus on certain measures on continuing education such as internal courses on palliative care, end-of-life communication, advance directives and advance care planning. Measures on continuing education were considered important for deepening knowledge of PPC and thus increasing the quality of PPC provision. The importance of communication about patients’ wills and end-of-life wishes was highlighted in a previous study by our research team (2020) [22]. However, in the present study, our expert group expressed strong criticism of the feasibility of these measures, citing heavy workloads, a lack of time and limited personnel as the main obstacles. These findings support a study by Seckler et al. (2020), showing that a lack of time, space and (educated) staff are the main reasons for failure in the implementation of such new measures [36]. International studies provide further evidence that measures are more likely to be implemented when they overlap with existing routines [36–38].

One half of our participants has already completed a certain training in palliative care and might put more emphasis on further education in that field. GPs without a qualification for palliative care might have felt overstrained with the implementation due to a lack of experience. Although some measures on continuing education were not included in the final intervention package, they provide a valuable basis for further development, taking the aforementioned studies into consideration. As a result, they have the potential to integrate continuing education into GP practices and thus increase the expertise of GPs and their teams. This would likely improve PPC provision and enhance the quality of life of patients receiving PPC.

In general, our participants reflected and complained about heavy workloads and a lack of time. A possible approach to help GPs prioritize certain tasks in their GPs office could be structured programs for change management. This could be essential to understand, that investing some time now has the potential to save time in the future.

### Strengths and limitations of the study

The main strength of the current study is the consensus of measures via the Delphi technique, based on expert opinions and suggestions [39]. As a result, the measures may be considered highly relevant and feasible for daily GP practice. In addition, participants’ socioeconomic background was highly representative of GPs in Germany. Nevertheless, we included a rather male, experienced and older group of GPs in our survey, half of whom have obtained a certification in palliative care, which might be source of bias in favour of special interest and knowledge in palliative care.

A limitation of the Delphi study is the missing perspective of other PPC providers and recipients. However, the views of these parties were integrated through participation action research in previous project steps, which informed the development of the recommended measures. The low participation rate and high dropout rate of GPs in the current study may be an indicator for limited interest in the field of palliative care and a discomfort with this topic. It further mirrors the limited time and staff resources of the participating GPs. These numbers are comparable to those reported in other studies conducted with GPs [40]. Furthermore, a positive selection bias might have been present in the group of Delphi respondents, and we cannot comment on the characteristics of non-responders. The extent of measure acceptance in other GP offices is unknown.

### Implications for practice

Now that the basic scientific work is done, the resulting measures and the according materials could be used in a larger number of GP offices to further improve the provision of PPC by GPs in practice. GP offices that use this intervention package could be recruited for future outcome research, also with patients and FCs. The use of our measures in collaboration with and led by the primary care professionals themselves could lead to valuable feedback to the research team.

To address experts’ most frequently cited challenges to improving PPC provision – lack of time, space and staff – further legislation pertaining to health care structures and payment reform may be required.

## Conclusion

The measures consented here provide valuable support for improving the integration of PPC into everyday GP practice. The measures can be used free of charge and adapted for use according to the needs of individual GP teams [ https://www.mhh.de/fileadmin/mhh/allgemeinmedizin/downloads/weitere_Downloadelemente/AllgMed_ALLPRAX_Interventionspaket.pdf]. This should support an expansion of PPC supply and an overall improvement in the quality of PPC provision by GPs.

## Supplementary Information

**Additional file 1.**

## Data Availability

All datasets used and/or analysed during the current study are available from the corresponding author upon reasonable request.

## Abbreviations

ALLPRAX: “Primary Palliative Care in General Practice”
FC(s): Family caregiver(s)
GP(s): General practitioner(s)
PPC: Primary palliative care
