# Supplementary material for: Improving primary palliative care – a Delphi consensus study on measures for general practice in Germany
Source: BMC Prim Care. 2022 Jan 17;23:12. doi: 10.1186/s12875-021-01613-7 (PMC8762944; doi:10.1186/s12875-021-01613-7)
Supplement: Supplementary file 1 — Additional file 1. [file 12875_2021_1613_MOESM1_ESM.docx]

After the fırst Delphi round, 11 out of 26 measures achieved consensus. 9 of the remaining 15 measures achieved consensus in the second round. In the end, 6 measures did not reach consensus. In this online appendix, we illustrate the percentages for relevance and feasibility, comments from the participants and changes in wording that we applied after each round for each measure.

**Measures that achieved consensus in the first round** (see Table 3)

In the first round, 11 out of the 26 measures achieved consensus. With respect to the consented measures, some additional suggestions were collected: Experts expressed that palliative care ‘crisis sheets’ (M4), which record PPC patients’ preferences for emergency situations, should use common language so they can be easily understood by all care providers. Participants also emphasised the importance of placing telephone numbers for emergency contacts and proxies (as selected by the patient) close to the patient’s bedside, as well as saving this information in the practice’s computer system (M6). N=9 experts pointed out a possible lack of experience amongst doctors on standby duty outside of regular practice hours (M10) and suggested that patients receiving palliative care have access to a more specialised emergency network. Furthermore, although having physician assistants refill home visit bags (M11) immediately after home visits (to prepare for upcoming palliative care home visits) reached consensus in the first round, n=4 GPs preferred to refill these bags themselves. Finally, difficulties during registration of all patients entering the practice (e.g. having cough) by physician assistants in the GP office (M16) (referring to, e.g., patient concerns and data privacy) were underlined.

**Measures that achieved consensus in the second round** (see Table 3)

In the second round, 9 of the remaining 15 measures achieved consensus. In round 1, the measure that a single physician assistant in the GP office should be made responsible for the coordination of patients receiving PPC and administering a structured first palliative care assessment (M1) on e.g. symptoms, problems and medication, was rated low in relevance (64%) and feasibility (57.4%). N=13 out of 64 GPs disapproved of the delegation of this responsibility to a single individual, citing problems due to limited and part-time staff capacity. Thus, the clear allocation of responsibility to a single physician assistant was removed from the measure’s wording for the second round, resulting in consensus with 85.3% relevance and 76.4% feasibility.

The development of an emergency dataset (M2) containing e.g. family caregiver contact data, was considered relevant (78.2%) in round 1. However, the experts criticised the feasibility (55.6%) of this measure, mainly because it involved saving medical data on a not-yet-existing electronic health care card. A further criticism pertained to the limited time resources of GPs and their physician assistants. After removing the electronic health care card from the measure’s wording for the second round, the measure achieved consensus with 97% relevance and 84.9% feasibility.

Use of a structured tool (e.g. the Supportive and Palliative Care Indicators Tool [1]) to identify patients who might benefit from PPC (M3) achieved 68.8% relevance and 75.8% feasibility in the first round. The expert group underlined the importance of personal experience but regarded structured guidelines a helpful addition. After including the resource of personal experience into the measure, consensus was achieved with 81.8% relevance and 78.8% feasibility.

In round 1, the sole use of leaflets about palliative care (M7) and the sole use of leaflets about advance directives and/or health care proxies (M8) achieved low relevance (70.3% for M7; 71% for M8) and moderate feasibility (83.9% for M7; 79% for M8). Experts preferred direct communication and considered leaflets a complementary resource. After modifying the wording of these measures accordingly, both relevance (85.3% for M7; 100% for M8) and feasibility (85.3% for M7; 97.1% for M8) achieved consensus in the second round and both measures were included in the final intervention package.

GPs further emphasised their sense of responsibility for the organisation and coordination of health care for patients receiving PPC (M12), underlining the importance of including all physician assistants in this task during joint consultations. Additionally, n=3 out of 64 experts suggested further cooperation with palliative care support centres or palliative care teams for more complex patients, as these institutes deliver specialised palliative care and can support GPs with their expertise [2]. After including these suggestions in the second round, consensus was reached with 88.2% relevance (68.8% in round 1) and 85.3% feasibility (68.8% in round 2).

In round 1, the measure that physician assistants should be responsible for updating all patients’ medication plans (M13) was heavily criticised in terms of relevance (52.3%) and feasibility (47.4%). N=18 out of 64 GPs argued that the responsibility for changes to the medication plan lay with doctors, who should review the indications for each medication regularly (n=1). Furthermore, the experts criticised the measure for its lack of focus on patients receiving PPC. After shifting the responsibility for updating the medication plan to GPs and adding a focus on patients receiving PPC, the measure achieved consensus in round 2, with 97.1% relevance and 97% feasibility.

The measure of providing physician assistants an undisturbed environment (e.g. a back office) for documentation during working hours (M17) achieved 90.6% relevance in the first round. However, time and space constraints resulted in low feasibility (59.7%). After the precondition of sufficient spatial capacity was added in round 2, the measure increased relevance (97.1%) and feasibility (82.4%); however, the experts still expressed doubts with respect to expanding office space.

The relevance of using a standardised fax template (M19) for admission requests to inpatient hospices or palliative care units was criticised in the first round (67.2%). However, feasibility was rated high (80.4%). The experts suggested that the template should include additional space to record individual information and, where necessary, follow-up contact via telephone should be initiated to discuss the takeover of a patient. After incorporating these suggestions in the second round, relevance (91.2%) and feasibility (85.3%) achieved consensus.

**Measures that did not achieve consensus** (see Table 3)

In the second round, 6 of the initial 26 measures did not achieve consensus.

Two measures pertaining to continuing education courses for the practice team on important PPC topics (M22: palliative care and end-of-life communication; M23: guidance on advance directives and advance care planning) were assessed as highly relevant in round 2 (M22: 87.9%; M23: 76.4%). However, time and staff constraints due to high workloads and a lack of offers for external courses on palliative topics were the experts’ main contra arguments, generating low feasibility ratings (M22: 69.7%; M23: 67.6%). Furthermore, the GPs did not agree on the relevance (round 1: 63.5%; round 2: 64.7%) or the feasibility (round 1: 50.8%; round 2: 51.5%) of making internal courses accessible to cooperating care providers and/or interested parties, such as home health care services (M14). In particular, a lack of time and space in GP offices was raised as a limiting factor.

GPs assessed the measure of being accompanied by physician assistants on home visits to patients receiving PPC (M24) as relevant (round 1: 78.1%; round 2: 91.2%) for improving their team’s hands-on practical experience in palliative care. However, staff and time constraints resulted in low feasibility (round 1: 61.1%; round 2: 70.6%). Furthermore, one expert raised that hands-on palliative care experience is already incorporated in the training for specialised medical care assistants.

The measure that physician assistants partake in an internship with a specialised palliative care provider (e.g. palliative care unit or inpatient hospice) (M25) was rated very low in both relevance (57.2%) and feasibility (31.2%) in round 1. N=6 GPs stated that they could not do without a physician assistant for an extended period of time, and suggested a maximum of one week for this internship. In addition, they pointed out that financing this time off would be challenging. After taking their feedback into account for the second round, relevance increased (85.3%) but feasibility (46.9%) was not achieved. Experts’ main argument referred to the imbalance between the number of patients receiving PPC and the number of patients without palliative care needs in the GP office.

Although participants agreed on the relevance of a medical information handover between two GPs prior to a temporary substitution (M26) (round 1: 90.6%; round 2: 97%), they did not agree on its feasibility (round 1: 64.5%; round 2: 73.5%). The experts criticised the coordination of joint home visits, as suggested in round 1, regarding them as too time-consuming. One expert suggested that a phone call would be more appropriate and another expert advocated for the development of a specific interface to simplify communication about palliative care patients.

**Literature**

1. Afshar K, Feichtner A, Boyd K, Murray S, Jünger S, Wiese B, Schneider N, Müller-Mundt G: Systematic development and adjustment of the German version of the Supportive and Palliative Care Indicators Tool (SPICT-DE). BMC Palliative Care 2018, 17(1):27.

2. Informationen für Hausärzte zum Thema SAPV. https://palliativnetz-peine.de/wp-content/uploads/2016/09/Informationen-für-Hausärzte-zum-Thema-SAPV.pdf. Accessed 19th February 2021.
